# Challenges and Solutions in Applying Large Language Models to Guideline-Based Management Planning and Automated Medical Coding in Health Care: Algorithm Development and Validation

**DOI:** 10.2196/66691

**Published:** 2025-11-10

**Authors:** Peter Sarvari, Zaid Al-fagih, Alexander Abou-Chedid, Paul Jewell, Rosie Taylor, Arouba Imtiaz

**Affiliations:** 1 Rhazes AI, 85 Great Portland Street, London, W1W 7LT, United Kingdom, 44 7762219374; 2 Assuric, London, United Kingdom; 3 Frimley Health NHS Foundation Trust, Camberley, United Kingdom

**Keywords:** AI assistant, large language model, LLM, GPT-4, retrieval-augmented generation, RAG, generation-assisted retrieval-augmented generation, GARAG, generation-assisted vector search, GAVS, medical informatics, digital health, generative AI in medicine, medical web application, automated medical coding, AI diagnosis, artificial intelligence

## Abstract

**Background:**

Diagnostic errors and administrative burdens, including medical coding, remain major challenges in health care. Large language models (LLMs) have the potential to alleviate these problems, but their adoption has been limited by concerns regarding reliability, transparency, and clinical safety.

**Objective:**

This study introduces and evaluates 2 LLM-based frameworks, implemented within the Rhazes Clinician platform, designed to address these challenges: generation-assisted retrieval-augmented generation (GARAG) for automated evidence-based treatment planning and generation-assisted vector search (GAVS) for automated medical coding.

**Methods:**

GARAG was evaluated on 21 clinical test cases created by medically qualified authors. Each case was executed 3 times independently, and outputs were assessed using 4 criteria: correctness of references, absence of duplication, adherence to formatting, and clinical appropriateness of the generated management plan. GAVS was evaluated on 958 randomly selected admissions from the Medical Information Mart for Intensive Care (MIMIC)–IV database, in which billed *International Classification of Diseases, Tenth Revision* (*ICD-10*) codes served as the ground truth. Two approaches were compared: a direct GPT-4.1 baseline prompted to predict *ICD-10* codes without constraints and GAVS, in which GPT-4.1 generated diagnostic entities that were each mapped onto the top 10 matching *ICD-10* codes through vector search.

**Results:**

Across the 63 outputs, 62 (98.4%) satisfied all evaluation criteria, with the only exception being a minor ordering inconsistency in one repetition of case 14. For GAVS, the 958 admissions contained 8576 assigned *ICD-10* subcategory codes (1610 unique). The vanilla LLM produced 131,329 candidate codes, whereas GAVS produced 136,920. At the subcategory level, the vanilla LLM achieved 17.95% average recall (15.86% weighted), while GAVS achieved 20.63% (18.62% weighted), a statistically significant improvement (*P*<.001). At the category level, performance converged (32.60% vs 32.58% average weighted recall; *P*=.99).

**Conclusions:**

GARAG demonstrated a workflow that grounds management plans in diagnosis-specific, peer-reviewed guideline evidence, preserving fine-grained clinical detail during retrieval. GAVS significantly improved fine-grained diagnostic coding recall compared with a direct LLM baseline. Together, these frameworks illustrate how LLM-based methods can enhance clinical decision support and medical coding. Both were subsequently integrated into Rhazes Clinician, a clinician-facing web application that orchestrates LLM agents to call specialized tools, providing a single interface for physician use. Further independent validation and large-scale studies are required to confirm generalizability and assess their impact on patient outcomes.

## Introduction

### Health Care’s Diverse Challenges

Health care is facing profound challenges that urgently require innovative solutions. Medical errors [1], overwhelming administrative burdens [2], understaffing [3,4], spiraling costs [5], and clinician burnout [6,7] threaten the foundations of medical care. Despite this, the health care sector has lagged behind other industries in adopting new technologies.

To address the above challenges, a subset of the authors has developed an AI-powered web app called Rhazes that helps doctors with paperwork and analytical tasks along their clinical workflow. Rhazes, like many other digital health startups, aims to offer integrated tools to health care professionals to match many of the growing needs in health care worsened by a staffing crisis [8-10]. AI-powered tools have been shown to have the potential to automate rote tasks [11], reduce errors [12,13], cut costs for everyone [14], improve clinician well-being and their patient-centeredness [15], and ultimately deliver better patient outcomes [12,16]. However, significant barriers and challenges persist in ensuring the safe and effective integration of AI within health care systems globally [17-19].

### Diagnostic Errors

Diagnostic errors in health care are more common than generally recognized and often receive less attention in both clinical practice and research. There are 2 ways to estimate their prevalence: autopsies and expert opinions. Various studies suggest a range between 5% and 20% [20-22], with the actual figure likely falling somewhere in between. Considering the 1.3 billion health care visits annually in the United States as an example, this percentage translates to a staggering 65 to 260 million diagnostic errors each year in the United States alone [23]. The rate of false negatives varies widely, from as low as 2.2% for myocardial infarction to an alarming 62.1% for spinal abscesses [20]. The National Academy of Medicine underscores the severity of this issue, attributing diagnostic errors to approximately 10% of patient deaths and 6% to 17% of hospital complications, with vascular events, infections, and cancers constituting around 75% of the serious harms from these errors [24].

Diagnostic errors arise from several causes. No-fault errors, such as those due to atypical disease presentations, are difficult to prevent. System-related issues, including delays in testing and communication failures, also play a role, though comprehensive data are limited. Cognitive errors, however, are the most frequent, with Graber et al finding that cognitive factors—such as faulty perception, failed heuristics, and cognitive biases—contribute to 74% of diagnostic errors [25]. This suggests that a substantial proportion of these errors could be mitigated through targeted interventions aimed at clinical decision-making and judgment. In malpractice claims, nearly 90% involve failures in these domains, regardless of the underlying condition [26].

### All Roads Lead to Burnout

Clinicians are responsible not only for making diagnoses but also for managing patient care thereafter. This process involves 3 major challenges. The first is staying up to date with a rapidly expanding medical knowledge base, which is estimated to double every 73 days [27] alongside frequently updated clinical guidelines. To address this, many physicians rely on peer-reviewed online clinical guideline databases, such as subscription-based services including UpToDate [28], DynaMed [29], as well as freely available resources like StatPearls [30]. The second challenge is identifying the most relevant guideline for a specific clinical scenario. The third, and often most complex, is tailoring these guidelines to the unique needs of each patient, taking into account individual characteristics, medical history, preferences, and socioeconomic context.

Medical errors often occur within the broader context of systemic pressures. One major factor is the administrative workload placed on clinicians. Studies indicate that for every hour spent in direct patient care, physicians spend approximately 2 additional hours on documentation and other administrative tasks [31]. This environment can contribute to situations where clinical duties become secondary to administrative responsibilities.

In the United States, more than half of physicians report at least 1 symptom of burnout [32], representing an estimated annual economic cost of US $4.6 billion or US $7600 per physician [33]. In the United Kingdom, physician burnout rates reached a record high in 2021 according to the annual national training survey [34].

### Medical Coding

Medical coding has clinical, statistical, and billing-related usages. Systematized Nomenclature of Medicine–Clinical Terms (SNOMED-CT; maintained by the International Health Terminology Standards Development Organization) is a terminology that provides clinicians with precise patient-specific information, including symptoms, diagnoses, procedures, and social contexts [35]. In the National Health Service (NHS), UK, SNOMED-CT is used for clinical coding, specifically to safely and accurately exchange information between health care providers. It is recorded at the point-of-care level and integrated into electronic health records (EHRs) as required by Fast Health Care Interoperability Resources [35], a health care data sharing standard. Most general practitioner clinics employ medical coders to translate patient findings into a mix of SNOMED-CT and in-house diagnostic codes for the most common cases. SNOMED-CT allows for more precise coding, as it not only comprises over 340,000 clinical [36] and 1.4 million drug-related codes but also describes the relationship between these codes, essentially functioning as an ontology [35]. In the United Kingdom, diagnostic codes using the *International Classification of Diseases, Eleventh Revision* (*ICD-11*) standard (published by the World Health Organization) and procedural codes using the Operating Procedure Codes Supplement (OPCS; published by NHS England) standard are recorded after the clinical event for statistical purposes, whereas in the United States, *ICD-11* is used for mainly billing purposes [37]. For coding procedures in the United States, the Health Care Common Procedure Coding System (HCPCS; published by the Centers for Medicare and Medicaid Services) is used. It has 2 levels: level 1 comprises Current Procedural Terminology (CPT) codes (published by the American Medical Association), which is used to bill for procedures done by health care professionals, and level 2 can be used to bill for products, supplies, and services used outside the physician’s office such as ambulatory services or orthotics [38]. Automated medical coding is needed for 2 main reasons: one is accuracy and the other is efficiency: the average coding accuracy is around 80% [37], with 83% in the United Kingdom and 89% in Scotland [39], and just the coding of backlog cases can take anywhere from several months to over a year [40].

### Large Language Models Could Help

Given the recent progress in artificial intelligence (AI), it has been proposed to help with various aspects of clinical work, including scribing and diagnosis [13,41,42]. GPT-4, a large language model (LLM) developed by OpenAI, has shown promise in medical applications with its passing of the medical board exam in multiple countries and languages [43-45]. A peer-reviewed study assessing the diagnostic ability of GPT-4 and Pathways Language Model 2 on 1000 electronic patient records reported that GPT-4 achieved a 93.9% diagnostic hit rate (lower bound), validated by 3 medical doctors [46]. Furthermore, the authors found that a quick and accurate automated diagnostic evaluation may be possible by presenting the ground truth data to GPT-4 and asking it to assess the diagnostic predictions made by LLMs [46,47]. This can then be used to rapidly benchmark different models and prompting strategies. A report published by OpenAI and Penda Health [48] claimed that AI Consult, a tool powered by LLMs, reduced diagnostic errors by 16% and treatment errors by 13% for 39,849 patient visits in Kenya.

When it comes to management planning, LLMs can revolutionize medical search and find recommendations for a specific clinical scenario by automatically citing the relevant guidelines. Examples include recently developed online medical search tools such as Elsevier Clinical Key [49] and MedWise [50]. The next level of automation is AI analyzing and extracting the relevant details from the EHR to adapt the clinical guidelines to the unique needs of the patient, essentially crafting a personalized treatment plan. An application capable of doing this is called a clinical decision support (CDS) [51] system, and industry examples of such tools include Glass Health [52] and Rhazes [53].

Another important application of LLMs in clinics is notetaking. Automated documentation leveraging ambient listening has shown promise in reducing clinician burden and improving the experience of doctor–patient interactions for both parties [54,55]. In addition, clinical evaluation of existing scribing tools such as Tortus [56], DeepScribe [57], Nuance Dragon Ambient Experience (DAX) [58,59], and Rhazes [60] has indicated enhanced documentation quality [56], increase in billed diagnostic codes, and potential time and cost savings [57,58,60]. However, such tools can cost US $1850 per month per clinician [58] and cause a worsening of after-hours electronic health records (EHR) usage [59]. In fact, Haberle et al found that Dragon Ambient Experience did not benefit documentation, productivity, or even patient experience but helped with provider engagement [59]. Ma et al argue that ambient AI scribes can even reduce time spent on the EHR, but further studies are needed to identify the users benefiting most from such technology [61].

Even though computer-assisted medical coding has been shown to improve coding accuracy [62], automating the clinical coding system appeared out of reach prior to the generative AI revolution due to technological and implementation-level challenges [63]. Non-LLM–based encoder–decoder-type models were shown to really struggle with identifying less frequent codes [64]; however, retrieval-augmented generation (RAG)–enhanced LLMs were recently found to be preferable to provider coders in terms of coding accuracy [65]. Generative AI seems to have made a big contribution toward the full automation of medical coding, and while we found no peer-reviewed evaluation paper to date, the authors of the previously cited paper, affiliated with Corti AI [66], who were researching non-LLM-based methods [64], are now leveraging generative AI to automate medical coding [66].

## Methods

### Ethical Considerations

The Medical Information Mart for Intensive Care (MIMIC)–IV [67] is a publicly available database and was previously ethically approved by the institutional review boards at Beth Israel Deaconess Medical Center (2001P001699) and the Massachusetts Institute of Technology (0403000206) in accordance with the tenets of the Declaration of Helsinki. The waiver of the requirement for informed consent was included in the institutional review board approval, as all protected health information was deidentified [67]. One of the authors (PS) was granted access to the database after completing training in human research (CITI Human Research certification number: 54889098) and signing a data use agreement in PhysioNet (agreement number 64081). The experiments described in this paper were conducted on Microsoft Azure (Azure OpenAI service) according to the “Responsible use of MIMIC data with online services like GPT” guidance by PhysioNet [68]. The code associated with this publication has been shared in an open repository, and information is provided in the “Data Availability” section of this manuscript.

### Generation-Assisted Retrieval-Augmented Generation for Clinical Decision Support

Here we demonstrate how to build a prototype for an AI-driven CDS, in particular, for crafting patient-specific management plans with verifiable citations from StatPearls [30], a point-of-care medical database with peer-reviewed clinical guidelines. On March 2, 2025, a comprehensive archive of clinical guidelines from StatPearls (approximately 1.5 gigabytes in size) was downloaded for use as the reference corpus in the RAG process. After unpacking the archive, a total of 9559 nxml files were obtained, each corresponding to the management of a distinct medical condition. These files were subsequently cleaned to remove nonclinical and extraneous information, including licenses, credits, warranties, publishing details, user prompts (eg, “Comment on this article”), and reference sections, so that only clinically relevant content remained. The title of each file was automatically inferred from the text and used as a filename, thereby linking each document to the medical event it described. The cleaned files were then converted into plain text format and uploaded to Azure Blob Storage. For citation purposes, a mapping was preserved between each inferred filename and the original download URL from StatPearls.

To enable semantic search and retrieval, the corpus was indexed within Azure Search Service. The indexing pipeline comprised a data source connection to Azure Blob Storage, a search index with fields for filename, chunk identifier, chunk text, and embedding vector; a text-splitting skill with a maximum chunk size of 4000 tokens and an overlap of 100 tokens; and an embedding skill using text-embedding-3-large model, OpenAI’s latest and best embedding model to date [69]. An indexer was then executed to vectorize and index the entire collection of document chunks.

**Figure 1.**
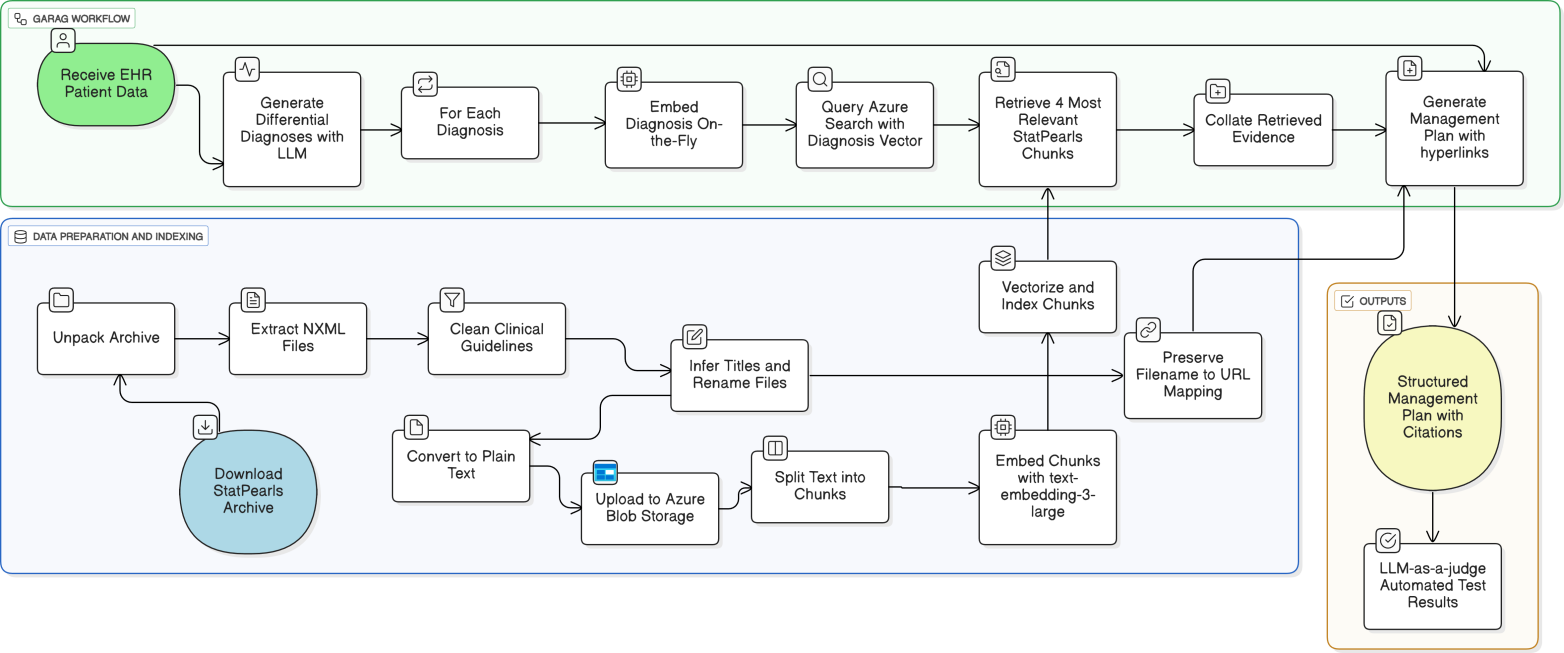
Generating a personalized evidence-based management plan using StatPearls and the GARAG framework. EHR: electronic health record; GARAG: generation-assisted retrieval-augmented generation; LLM: large language model.

Building upon this foundation, we developed a proof-of-concept workflow, which we termed generation-assisted retrieval-augmented generation (GARAG). GARAG proceeds in 3 stages. First, given EHR data, an LLM (specifically GPT-4.1) is prompted to generate a structured list of differential diagnoses. Second, for each diagnosis, the system queries the indexed StatPearls corpus through Azure Search Service, employing the Hierarchical Navigable Small World approximate nearest neighbor algorithm (with parameters M=4, efConstruction=400, and efSearch=500) and cosine similarity as the distance metric. The 4 most relevant text chunks are retrieved for each candidate diagnosis. Third, the LLM is prompted again with the patient data and the retrieved evidence sources. From this input, the model generates a structured management plan covering investigations, treatment suggestions, supportive management, other considerations, risks, and references. Citations are automatically hyperlinked to the original StatPearls sources via the preserved filename-to-URL mapping (Figure 1). A key advantage of GARAG is its ability to ground recommendations in guideline-specific, peer-reviewed sources tailored to each predicted diagnosis. This targeted retrieval avoids the information dilution that can occur with standard RAG approaches, where embedding the entire case may obscure fine-grained clinical details. By structuring the workflow around diagnosis-specific guideline retrieval, GARAG ensures that management plans are directly aligned with authoritative clinical references.

For clarity, we have provided a Python Jupyter Notebook that demonstrates our implementation of such a GARAG system and the automated tests we have run to confirm that the instructions are being followed by the LLM. To contrast the GARAG workflow to a traditional RAG workflow, we also provide the reader with a Python prototype that generates treatment plans with StatPearls references using RAG.

### Generation-Assisted Vector Search for Automated Medical Coding

Next, we demonstrate how to build an AI tool for automated medical coding using a method we named generation-assisted vector search (GAVS). Unlike RAG, in which retrieval precedes generation, GAVS inverts the process: generation is performed first and retrieval follows. In this pipeline, an Azure OpenAI LLM is few-shot prompted to read EHR text and to enumerate, with maximal granularity, the clinically relevant entities for coding (eg, diagnoses/comorbidities/abnormalities, procedures/treatments/services, and drugs). Each generated item is subsequently embedded with text-embedding-3-large and matched against a vector database representing the target coding ontology.

For procedural coding, the coding ontology was derived from the official 2025 CPT release downloaded as an Excel workbook from the Centers for Disease Control and Prevention government website. The worksheet containing all CPT entries (“ALL 2025 CPT Codes”) was parsed to retain the canonical code identifier and its short description; rows with missing codes were removed. Each row was converted into a LangChain [70] Document whose embedding encodes the description, while the metadata preserves both the CPT Code and Title. Because CPT descriptions are short, no chunking was required.

Embeddings were stored in a Pinecone serverless index [71] and queried via the LangChain [70] PineconeVectorStore at runtime. During inference, each model-generated item (eg,procedure) in a structured list output is embedded on the fly and used to perform semantic search, retrieving the top 10 nearest CPT entries by cosine similarity. The returned results include both semantic scores and the canonical CPT codes via the stored metadata, allowing the system to report human-readable candidates (code+title) alongside each extracted clinical item.

The same pattern generalizes to diagnostic and pharmacological coding by substituting the target ontology (eg, *ICD-11* or SNOMED CT for diagnoses; SNOMED CT for drugs) and constructing an analogous vector store with description-level embeddings and code identifiers preserved in metadata. For transparency and reproducibility, we provide a Jupyter Notebook demonstrating the full CPT workflow end-to-end, including data acquisition, runtime index creation, LLM-based generation, and similarity search-based mapping. Crucially, the index is created and populated programmatically at runtime if absent (index name cpt-cdc-2025-text-embedding-3-large, dimension 3072, metric cosine, region eu-west-1); if present, the pipeline connects to the existing index without reingestion. We also provide links to download coding ontologies from the official publisher websites in the format of single or multiple Excel files: CPT from the Centers for Medicare and Medicaid Services [72], *ICD-11* from the World Health Organization [73], and SNOMED from the NHS Digital website [74].

To quantitatively assess the benefits of GAVS over direct LLM prompting, we conducted a proof-of-concept evaluation for *International Classification of Diseases, Tenth Revision* (*ICD-10*) coding following the methods of Sarvari and Al-Fagih [47]. We initially sampled 1000 admissions at random from MIMIC-IV, of which 42 did not have officially assigned *ICD-10* codes and were excluded, resulting in a final cohort of 958 admissions. For each admission, the set of billed *ICD-10* codes served as the ground truth. Across this cohort, there were 8576 total *ICD-10* codes at the subcategory (full code) level, comprising 1610 unique subcategories. When mapped to parent categories, the total number decreased to 7311 codes across 540 unique categories. The discrepancy (8576 vs 7311) reflects cases in which the Python library used for mapping [75] did not recognize certain overly specific subcategory codes, in which case no parent category was assigned. The full *ICD-10* ontology includes 95,109 valid codes, defining the candidate space for prediction. Two approaches were compared. In the vanilla LLM method, GPT-4.1 was prompted directly to predict *ICD-10* codes for each admission, without external constraints. In the GAVS method, GPT-4.1 was first prompted to generate granular diagnostic entities, which were then embedded and matched to the top 10 most similar *ICD-10* codes through vector search (Azure Search Service) of the official ontology. Both methods used identical LLM configuration and dataset preprocessing, ensuring comparability across experiments. The primary outcome was recall, defined as the fraction of ground-truth codes correctly predicted. Two variants were calculated, consistent with [46] (1) average (per-admission mean) recall, averaged across the 958 admissions, and (2) aggregate (weighted average) recall, defined as the total number of correctly predicted codes divided by the total number of codes (8576) or 1−(∑missed codes across admissions / ∑true codes across admissions).

Precision was not reported, as discussed previously [47], because billing records are not a reliable gold standard for false positive determination: clinically valid diagnoses often go unbilled, and multiple codes may be acceptable matches (especially when working with incomplete data). In this context, precision metrics would therefore be misleading. For statistical comparison, following [47], we applied a 2-proportion *z* test to evaluate differences between methods in recovered versus missed ground-truth codes.

For clarity, we provide the reader with a Python Jupyter notebook, demonstrating the entire automated coding prediction (including the vanilla GPT-4.1 and GAVS methods for predicting *ICD-10* codes) and evaluation workflow both at the subcategory and category levels.

## Results

### GARAG: Citation Integrity and Relevance

Using the LLM-as-a-judge method [47,76], we evaluated the GARAG workflow on 21 clinical test cases created by a subset of the authors who are medical professionals. Each case was executed 3 times independently to assess reproducibility, yielding a total of 63 runs. Performance was assessed using four criteria: (1) correctness of references, (2) absence of duplicate citations, (3) adherence to citation formatting standards, and (4) contextual appropriateness of the generated management plan, including whether it explicitly addressed the presented diagnoses. Across all 63 runs, 62 satisfied all 4 criteria, corresponding to a success rate of 98.4%. The single exception occurred in case 14 during its first repetition, in which references were accurate but displayed a minor ordering inconsistency, with “[3]” appearing before “[2].” Importantly, no spurious references were observed, all citations could be traced directly to their StatPearls sources, and all management plans were judged clinically relevant. These findings demonstrate that GARAG provides a highly reliable and reproducible workflow for generating clinical management plans with proper citation handling, with only minor formatting issues detected across repeated executions.

### GAVS: Diagnostic Coding Performance

We next evaluated the GAVS method for automated diagnostic coding on 958 randomly selected MIMIC-IV hospital admissions. Across these cases, there were 8576 total assigned *ICD-10* codes at the subcategory level, spanning 1610 unique subcategories. When collapsed to categories using a Python mapping library [75], this corresponded to 7311 total codes across 540 unique categories. The full *ICD-10* ontology contains 95,109 valid codes, underscoring the scale of the prediction task. Two approaches were compared: (1) a direct LLM baseline, in which GPT-4.1 was prompted to predict *ICD-10* codes without constraints, and (2) GAVS, in which GPT-4.1 first generated granular diagnostic entities that were each mapped onto the top 10 matching *ICD-10* codes through vector search over the official ontology. Across all admissions, the vanilla LLM produced 131,329 candidate codes, while GAVS produced 136,920. At the subcategory level, the vanilla LLM achieved a mean recall of 17.95% (15.86% weighted), whereas GAVS achieved 20.63% (18.62% weighted), representing a statistically significant improvement (*P*<.001, 2-proportion *z* test). Notably, GAVS generated 11,254 unique predicted subcategories, compared with 15,572 unique subcategories from the vanilla LLM, suggesting that the vanilla approach was more diffuse in its predictions, whereas GAVS concentrated predictions on a narrower and more relevant set of codes. At the category level, the vanilla LLM achieved a mean recall of 34.05% (32.60% weighted), while GAVS achieved 33.57% (32.58% weighted). The difference was not statistically significant (*P*=.99). GAVS produced 1192 unique predicted categories, compared with 913 unique categories for the vanilla LLM.

### Integration Into Rhazes Clinician

Building on the GARAG and GAVS prototypes, we developed a clinician-facing agentic web application that integrates documentation assistance, management planning (GARAG), automated coding (GAVS), and differential diagnosis tools and is accessible via the Rhazes website [53]. To maximize accessibility, the system was deployed as a Progressive Web Application, enabling installation and seamless use across desktop and mobile platforms without requiring a native app. The application was implemented using a modern web architecture: a Next.js full-stack framework with React (TypeScript) for the front end, a Node.js backend, and a PostgreSQL database accessed through the Prisma object-relational mapper. Hosting was provided on Aptible [77], a platform-as-a-service offering secure, Health Insurance Portability and Accountability Act–ready infrastructure. Within Rhazes, user queries are handled by LLM agents that route requests to the most appropriate tools. These include the management planning (GARAG) and medical coding (GAVS) pipelines described above, a documentation assistant for completing predefined templates, and a differential diagnosis tool that was previously evaluated in Sarvari and Al-Fagih [47]. The orchestration layer was built on LangGraph [70], allowing for parallel tool execution and a persistent shared conversation history across agents. The system, certified under Cyber Essentials [78,79], is used by thousands of doctors and supports integration with major EHR systems, including Epic (32.8% market share in 2021) and Cerner (23.2% market share in 2021) [80].

## Discussion

### Principal Findings

In this study, we introduced Rhazes, an AI assistant for doctors designed to handle paperwork and analytical tasks in clinical medicine. Rhazes aims to free physicians from the burden of documentation and to help them provide better care for more patients. Within this broader system, the GARAG and GAVS frameworks demonstrate the feasibility of embedding structured guardrails into LLM-based clinical workflows. GARAG ensures that management plans are grounded in peer-reviewed guideline sources with properly formatted references, achieved through a diagnosis-first retrieval workflow that increases the likelihood of relevant sources being cited. GAVS applies a similar principle to coding, improving fidelity through a 2-step process in which diagnostic entities are generated first and then deterministically mapped to valid ontology terms via vector search. Both methods represent proof-of-concept prototypes that were subsequently deployed within the Rhazes Clinician platform. Although GARAG was evaluated on a smaller case set compared with GAVS, its strength lies in preserving fine-grained diagnostic information during retrieval. By generating diagnoses first and then retrieving guideline evidence for each one, GARAG avoids the information dilution that occurs when the entire patient record is embedded at once, ensuring that management plans remain tightly linked to diagnosis-specific guidance.

Taken together, the evaluation results indicate that GAVS improves resolution at the subcategory level without sacrificing performance at the broader category level. Beyond this quantitative advantage, GAVS has 3 qualitative benefits that strengthen its reliability and scalability. First, GAVS guarantees that every predicted code is part of the official coding ontology. Because predictions are drawn directly from a vector search over the ontology, the system cannot hallucinate nonexistent codes—a risk that remains with unconstrained LLM outputs. Second, GAVS is flexible across coding systems. Adapting it to a different *ICD* version, or to CPT/SNOMED, or to institution-specific ontologies requires no retraining or prompt engineering. One simply replaces the vector database with embeddings of the target ontology’s code descriptions, and the method functions seamlessly. Third, GAVS enhances explainability. The LLM provides a structured list of diagnostic predictions together with textual reasoning, and each prediction is then mapped deterministically to a small, fixed set of candidate codes via cosine similarity. This 2-step design ensures systematic and interpretable outputs. By contrast, a vanilla LLM generates codes as a single sequence based on statistical likelihood, with no guarantee of coverage, ordering, or manageable length, making its reasoning harder to audit and its predictions less scalable.

### Future Work

There are many feature improvements we envisage adding to Rhazes Clinician soon. First, we plan to experiment with new embedding models such as Guided In-Sample Selection of Training Negatives-large-embedding-v0 [81], which has been identified as a good fit for clinical tasks in a previous study [82]. The change in the embedding model means that we will have to reindex the latest versions of the clinical and coding guidelines we have been using for GARAG and GAVS. From a platform perspective, Rhazes already supports *ICD*, CPT, and SNOMED codes. We plan to extend this support to the full HCPCS [38] (including level 2) as well as OPCS. These additions will broaden coverage across clinical and administrative workflows. From an evaluation perspective, future work will focus on systematically assessing whether GAVS’ advantage over an unconstrained LLM generalizes across coding ontologies beyond *ICD-10*. We will design blinded, head-to-head comparisons—similar in spirit to Klang et al [65]—spanning CPT, SNOMED, HCPCS level 2, OPCS, and clinic-specific ontologies, with physicians and LLMs independently adjudicating results. Because these ontologies (particularly the less common or locally maintained ones) are less likely to be represented in pretraining data, our a priori hypothesis is that the relative benefit of GAVS will be larger than what we observed for *ICD-10.* As part of this program, we aim to construct and share a deidentified, gold-labeled coding dataset suitable for benchmarking across methods. Additional methodological work will examine the effect of the vector-search candidate set size (eg, top-k), alternative embedding models [81,82], and improved parent-mapping resources to reduce unresolvable cases during category aggregation. Finally, we plan to extend our EHR integration offerings: we aim to support Egton Medical Information Systems, the leading EHR for UK primary care clinics, and SystmOne, the second most popular EHR for UK general practitioners [83]. These integrations will facilitate prospective, multisite evaluations and subgroup analyses while maintaining interoperability with existing clinical systems.

### Limitations

The GARAG workflow was tested on a relatively small set of 21 author-designed cases. While reproducibility was high, independent validation on larger and more varied case sets is needed. The GAVS evaluation, while based on a sizable cohort of 958 admissions, relied on billing records as the gold standard. Because billing data do not fully capture the clinical picture of each admission, it is not possible to definitively establish precision, as some well-reasoned diagnostic predictions may go unbilled [46,47]. Moreover, the underlying MIMIC-IV dataset has well-recognized constraints: it lacks clinical notes, physical examination findings, and certain test results such as electrocardiograms, and it is drawn from a single hospital in Boston, MA. This means the data are subject to demographic and institutional biases and may not generalize to other patient populations. Finally, some specific *ICD-10* codes could not be mapped to parent categories due to library limitations. These factors highlight the need for further testing against richer clinical datasets, across multiple institutions, and with more comprehensive ontology mappings. Taken together, these component-level limitations reflect broader challenges in deploying LLM-driven systems like Rhazes into clinical practice. The effectiveness of an AI co-pilot hinges on its accuracy across diverse clinical scenarios. In general, evaluation of clinical performance of LLMs is challenging due to the lack of transparency when it comes to versions, prompts, human evaluations, LLM-as-a-judge evaluations [47,76], patient data, and due to the nonexistence of gold-labeled data sets for many clinical applications [84]. Care must be taken to accurately assess AI for improved patient outcomes and to avoid statistically flawed evaluations [85]. In practice, AI tools can exhibit degraded performance when used outside the conditions of their training (out-of-distribution use). Even models that performed well in development or obtained regulatory clearance have underperformed in new settings due to poor generalization. This raises the risk of missed diagnoses or incorrect management plans if the AI encounters patient data that differs from its training distribution. Continuous validation of the system on local patient populations is therefore critical to ensure reliability in AI-generated recommendations [86]. AI models learn from historical data, so any biases or gaps in those data can lead to skewed or inequitable outcomes [87]. If the training dataset underrepresents certain demographics or conditions, the model suggestions may be less accurate for those groups, potentially perpetuating health disparities. For example, studies have found some clinical AI algorithms perform significantly worse for female patients or racial minorities, underdiagnosing these groups compared to others [88,89]. Such bias not only affects accuracy but also violates principles of fairness in care. Ensuring the data used by AI co-pilots are diverse and representative is essential to minimize this risk. We must also be mindful of other harmful biases LLMs may learn during training [84], as well as the risks that over-reliance on AI systems may bring to medicine (eg, automation bias) [90].

### AI in Health Care Ethics

Under the General Data Protection Regulation, health care organizations can often process patient data for care without explicit consent, provided they have a valid lawful basis (Article 6) and meet a special category condition (Article 9) [91], such as provision of health care services. This lawful basis should naturally extend to the data processors used, such as the AI scribes. However, alongside lawfulness, transparency is important. Patients should be informed when an algorithm is involved in their diagnosis or treatment planning. Research indicates that disclosing the use of an AI tool is essential to patients; a recent study found that patients strongly prefer to be informed when AI assists in their care and recommended that explicit consent for AI involvement be obtained during the clinical workflow [92]. In the context of Rhazes, this means clinicians should be transparent about the AI’s role—explaining to patients that an AI system will analyze their data and contribute to suggestions. Such transparency not only respects patient autonomy but also helps build trust, as patients are more likely to accept AI-derived recommendations if they understand and agree to their use.

An AI system must consistently uphold the core principles of medical ethics—beneficence, nonmaleficence, autonomy, and justice. One concern is that software like Rhazes might, in some situations, propose an option that, while data-driven, conflicts with a patient’s values or broader ethical norms. For example, an AI might prioritize treatments based on statistical outcomes or cost-effectiveness, which could unintentionally de-emphasize a patient’s personal preference for quality of life. If Rhazes recommends an aggressive treatment purely because it maximizes survival odds, but the patient prioritizes comfort, blindly following the AI would undermine patient autonomy. Human clinicians must interpret Rhazes’ outputs through the lens of their professional ethics and clinical judgment. They should override or adjust recommendations that do not fit the patient’s individual context or the ethical standards of care. In essence, Rhazes should support clinical decisions that are not only effective but also ethically sound, with the physician ensuring final decisions align with the principle of autonomy and patient-centered care. Several approaches can address these ethical concerns and ensure that AI tools are used responsibly in health care. One key strategy is incorporating explainability into the AI model. Rather than acting as a “black box,” Rhazes provides interpretable reasoning or an explanation for its suggestions (for instance, highlighting which patient factors or medical evidence led to a given diagnostic recommendation). Explainable AI methods help clinicians and patients understand why a recommendation was made, which is vital for trust and for verifying that the recommendation makes ethical and clinical sense. Another strategy is clinician oversight and accountability. Rhazes is intended to assist, not replace, the clinician; therefore, protocols should emphasize that the human provider retains ultimate responsibility for diagnosis and treatment decisions. By maintaining clear accountability—where the clinician must review and approve AI-generated plans—the risk of blind adoption of incorrect suggestions is reduced. Studies on automation bias mitigation have noted that training users and stressing their accountability can counter overreliance [93]. Regular training sessions for clinicians on the proper use of Rhazes, including case studies of when the AI errs, can sharpen their judgment on when to trust the AI and when to apply caution. Finally, patient education about AI in health care can help. Patients should be informed in understandable terms what Rhazes is and what role it plays in their care. When patients understand that the AI is a tool used by their doctor (and not a substitute for the doctor), it can alleviate fears of a purely machine-driven care plan. Surveys have shown that both doctors and patients feel anxious if they do not understand AI’s involvement [94], so educational efforts (leaflets, consent discussions, etc) can demystify the technology. In summary, through explainable AI design, strong human oversight, and educational transparency, Rhazes can be deployed in a way that upholds ethical standards and supports clinicians and patients alike.

### Compliance Requirements for AI Tools in Hospitals

Any digital health technology company operating within the United Kingdom collecting or processing any form of personal data must comply with UK General Data Protection Regulation and the Data Protection Act [95]. Companies processing personal data in the United Kingdom must be registered with the Information Commissioner’s Office (ICO) [96]. In addition to the usual requirements around processing personal data, it is likely that a health-tech company will be processing sensitive personal health data, which would classify as special category data. This can bring some additional requirements, such as the need or recommendation to complete a Data Protection Impact Assessment [97]. There must also be appropriate contracts and Data Processing Agreements [98] in place between an NHS organization and the digital health supplier, between which personal data may flow.

If a digital health supplier is looking to work with NHS organizations and will be interacting with NHS patient data, they will need to complete the Data Security and Protection Toolkit (DSPT) [99]. This is not a requirement in the private sector or in direct-to-consumer models. If an organization is an IT supplier with 50+ staff members and has a turnover of at least £10 million (US $13.16 million) and supplies digital goods and services to the NHS, the company must also undertake an independent audit/assessment [99]. Organizations handling patient data may require the following personnel: a data protection officer (DPO), a senior information risk owner, and a Caldicott guardian [99]. A DPO [100] is required if a company’s core activities consist of large-scale processing of special category data. DPOs help to monitor internal compliance, inform on data protection obligations, provide advice regarding Data Protection Impact Assessments and act as a point of contact for data subjects and the ICO. The Senior Information Risk Owner is a senior member of the organization whose roles are to promote a culture that values and protects ICO information, own information risk management policies and processes and ensure they are implemented, advise on information risk management processes and provide assurance, and own the incident management framework. The Caldicott Guardian [101] is a senior person responsible for overseeing the use and sharing of patient information by protecting the confidentiality of people’s health and care information.

Companies aiming to deploy in the NHS also must go through information security and technical assurance. UK Cyber Essentials [78,102,103] is a self-assessment that any company looking to work with the public sector must comply with. The general recommendation is for companies to comply with Cyber Essentials Plus, which involves both a self-assessment and an external audit. Often, companies will use ISO 27001 to demonstrate a higher level of security than required by just meeting DSPT requirements, but it is not generally mandated in health care organizations. ISO 27001 is an internationally recognized standard for information security [102] that is not health care specific. It requires companies to implement Information Security Management Systems and focuses on risk assessment. It requires independent certification by an accredited body. Digital health technologies deployed in NHS organizations in England will also need to comply with the digital clinical safety standards DCB0129/0160. This is required by law, under section 250 of the Health and Social Care Act 2012 [104]. Both the manufacturer (DCB0129) and deploying health or social care organization (DCB01060) are required to complete a clinical risk assessment, including key documentation. This process is overseen by an appropriately qualified Clinical Safety Officer. There is also a requirement to monitor and record any incidents post-deployment. Penetration testing is required to assess the security of digital health technologies deployed in NHS organizations as part of the NHS Digital Technology Assessment Criteria (DTAC) [105], which mandates that any identified vulnerabilities should be appropriately remediated. The NHS DTAC [105] is a framework that brings together legislation and best practice in 5 core areas: clinical safety, data protection, technical security, interoperability, and accessibility and usability, which incorporates the aforementioned NHS DSPT and DCB0129 standards. The DTAC is a national baseline criteria for digital health technologies being deployed within NHS health care organizations and can be used by health care organizations to assess suppliers as part of their due diligence process. Due to the complexity of navigating NHS compliance frameworks, specialized firms have emerged to help digital health companies accelerate clinical assurance processes; these include Assuric [106], Vanta [107], and Naq [108].

If the AI product meets the definition of software as a medical device (SaMD), companies would also need to comply with medical device regulation and achieve appropriate certifications before being available for use in the open market. This would be the case if the intended purpose and functionality of the product extends into diagnosis, prevention, monitoring, prediction, prognosis, treatment, or alleviation of disease, as defined by European Union (EU) Medical Device Regulation [109]. SaMD is defined as “software intended to be used for one or more medical purposes that perform these purposes without being part of a hardware medical device” [110]. In the United Kingdom, medical devices are classified by risk to class 1, class 2a, class 2b, and class 3, with class 1 being low risk to patients and class 3 being high risk to patients. Manufacturers face a greater scope of work and evidentiary burden when dealing with higher-risk products. In the United Kingdom, low-risk class 1 devices require manufacturers to make a self-declaration of conformity to the Medicines and Healthcare Products Regulatory Agency (MHRA). Other classes require involvement and approval from an approved body (an organization designated by the MHRA to assess the conformity of products before they are placed on the market), granting a UK Conformity Assessed mark, the equivalent to a Conformité Européenne (CE) mark in the EU.

There have been several AI-as-a-medical device products on the market for some time, primarily in the category of diagnostic radiology or dermatology tools, one example being Skin Analytics, which recently achieved regulatory approval for autonomous AI skin cancer detection system Deep Ensemble for Recognition of Malignancy in Europe, receiving class III CE marking. This is the first legally authorized AI to independently make clinical decisions on skin cancer without oversight. Deep Ensemble for Recognition of Malignancy achieves 99.8% accuracy rate in ruling out cancer, surpassing the performance of dermatologists who typically achieve 98.9% [111]. However, there is yet to be a generative AI product that has been certified as a medical device in the United Kingdom or EU. In the United States, Modella AI’s generative AI co-pilot, PathChat, has received device designation by the Food and Drug Administration. This is the first regulatory approval of a clinical-grade generative AI co-pilot [112] and is the first of likely many more SaMD generative AI applications.

The NHS 10-Year Reform Plan sets a clear direction for modernizing care delivery, with a strong emphasis on digital transformation, integrated community services, and reducing strain on clinical staff [113]. One area gaining significant momentum is the deployment of AI-enabled ambient scribing tools, which offer practical relief from administrative overhead by automatically transcribing and summarizing clinical encounters [113]. The plan explicitly highlights the need to streamline documentation and use responsible automation to release clinician time for patient care [113]. In parallel, NHS England’s technical guidance on ambient voice technology adds further clarity and outlines key regulatory considerations for these tools [114]. Pure transcription tools are generally not considered medical devices. However, where generative AI features extend into summarization, providing prompts, generating structured clinical notes, letters, or codes, they are likely to qualify as SaMD. Such tools would then require UK Conformity Assessed or CE marking, MHRA registration, and a full clinical safety case under DCB0129/0160. Beyond regulatory certification, NHS organizations are expected to ensure integration with existing EHRs through standards such as Fast Health Care Interoperability Resources, HL7, and SNOMED CT, maintain strong human oversight to mitigate diagnostic drift or foreseeable misuse, and implement a clear post-deployment monitoring framework. This includes mechanisms for clinicians to flag transcription errors, routine audits of scribe outputs, and attention to bias risks, particularly for patients with regional accents, dialects, or speech impairments.

### Conclusion

While AI has been rapidly evolving over the last 2 years, progress has not been reciprocated in the health care industry, a heavily regulated space with many financial, staffing, and quality-of-service-type problems. Due to the lack of gold-labeled datasets and human evaluation protocols for LLM-generated text, recent AI in health care innovations was driven by well-funded industry players who were able to start generating evidence by securing hospital pilots early. So far, most companies seek to innovate in administrative workflows that avoid direct patient care as this comes with lesser regulatory burden. There seems to be a regulatory gray area surrounding workflows which could ultimately affect patient care should doctors over-rely on AI. Examples include AI scribing and clinical document generation, with only a few AI notetaker tools evaluated in academic journals with often conflicting and lack of reproducible results.

In this article, we reviewed the need for AI tools in health care and the current state of the industry, including dominant players and their progress. During this review, we demonstrated, firsthand, how such tools may be created and how they may be used by physicians. We discussed key implementation considerations for Rhazes Clinician, an AI assistant for doctors. We described in detail the methods used to create a CDS and an admin assistant for doctors, including the LLMs deployed, the clinical guidelines used, the RAG hyperparameters, and the cloud services used. We also introduced a new method for medical coding that we dubbed GAVS for Generation-Augmented Vector Search and an improved RAG workflow for CDS that we named GARAG. GARAG highlights the value of diagnosis-specific retrieval, allowing management plans to stay closely linked to diagnostic evidence while avoiding the information dilution that occurs when entire patient records are embedded in typical RAG workflows. We showed that GAVS statistically significantly improves *ICD-10* coding predictions. For both treatment planning and medical coding, we provided Jupyter notebooks that demonstrate the (albeit simplified) implementation of these Rhazes tools. Our goal with this is to contribute to the academic discussion about AI tools for health care and encourage academics as well as industry players to share their datasets and novel methods in order to accelerate the deployment of transparent AI tools in hospitals.

## Abbreviations

AI: artificial intelligence
CDS: clinical decision support
CPT: Current Procedural Terminology
DPO: data protection officer
DSPT: Data Security and Protection Toolkit
DTAC: Digital Technology Assessment Criteria
EHR: electronic health record
GARAG: generation-assisted retrieval-augmented generation
GAVS: generation-assisted vector search
HCPCS: Health Care Common Procedure Coding System
*ICD-10*: *International Classification of Diseases, Tenth Revision*
*ICD-11*: *International Classification of Diseases, Eleventh Revision*
ICO: Information Commissioner’s Office
LLM: large language model
MHRA: Medicines and Healthcare Products Regulatory Agency
MIMIC: Medical Information Mart for Intensive Care
NHS: National Health Service
OPCS: Operating Procedure Codes Supplement
RAG: retrieval-augmented generation
SaMD: software as a medical device
SNOMED-CT: Systematized Nomenclature of Medicine–Clinical Terms
